# The Capsid Protein VP1 of Coxsackievirus B Induces Cell Cycle Arrest by Up-Regulating Heat Shock Protein 70

**DOI:** 10.3389/fmicb.2019.01633

**Published:** 2019-07-17

**Authors:** Yao Wang, Shuoxuan Zhao, Yang Chen, Tianying Wang, Chaorun Dong, Xiaoman Wo, Jian Zhang, Yanyan Dong, Weizhen Xu, Xiaofeng Feng, Cong Qu, Yan Wang, Zhaohua Zhong, Wenran Zhao

**Affiliations:** ^1^Department of Cell Biology, Harbin Medical University, Harbin, China; ^2^Department of Microbiology, Harbin Medical University, Harbin, China; ^3^Northern Translational Medicine Research Center, Harbin Medical University, Harbin, China

**Keywords:** coxsackievirus B, capsid protein VP1, cell cycle, G1 arrest, heat shock protein 70, heat shock factor 1

## Abstract

Manipulating cell cycle is one of the common strategies used by viruses to generate favorable cellular environment to facilitate viral replication. Coxsackievirus B (CVB) is one of the major viral pathogens of human myocarditis and cardiomyopathy. Because of its small genome, CVB depends on cellular machineries for productive replication. However, how the structural and non-structural components of CVB would manipulate cell cycle is not clearly understood. In this study, we demonstrated that the capsid protein VP1 of CVB type 3 (CVB3) induced cell cycle arrest at G1 phase. G1 arrest was the result of the decrease level of cyclin E and the accumulation of p27^Kip1^. Study on the gene expression profile of the cells expressing VP1 showed that the expression of both heat shock protein 70-1 (Hsp70-1) and Hsp70-2 was significantly up-regulated. Knockdown of Hsp70 resulted in the increased level of cyclin E and the reduction of p27^Kip1^. We further demonstrated that the phosphorylation of the heat shock factor 1, which directly promotes the expression of Hsp70, was also increased in the cell expressing VP1. Moreover, we show that CVB3 infection also induced G1 arrest, likely due to dysregulating Hsp70, cyclin E, and p27, while knockdown of Hsp70 dramatically inhibited viral replication. Cell cycle arrest at G1 phase facilitated CVB3 infection, since viral replication in the cells synchronized at G1 phase dramatically increased. Taken together, this study demonstrates that the VP1 of CVB3 induces cell cycle arrest at G1 phase through up-regulating Hsp70. Our findings suggest that the capsid protein VP1 of CVB is capable of manipulating cellular activities during viral infection.

## Introduction

The progression of cell cycle is a dynamic process in which proliferating cells divide into two daughter cells through the orderly events occurred at four stages, G1, S, G2, and mitosis (Maes et al., 2017). The control of cell cycle ensures the faithful replication and segregation of the genome (Fan et al., 2017). The unidirectional progression of cell cycle is controlled by the periodic activation of cyclin-dependent kinases (Cdks). The activity of Cdks largely depends on their partnership with specific cyclins in the distinct phase of cell cycle. In mammalian cells, cyclin D, cyclin A/E, and cyclin A/B couple with Cdk4/6, Cdk2, and Cdk1, respectively, to coordinate the transition between distinct cell cycle stages (Sanchez and Dynlacht, 2005).

Viruses are cellular parasites that replicate and assemble either in cytoplasm or in the nucleus. To sustain viral replication, the cellular environment must be manipulated to provide adequate raw materials for the biosynthesis of the virus (Schang, 2003; Ning et al., 2017; Torshizi et al., 2017; Wang Z.Y. et al., 2017; Wasson et al., 2017; Xu et al., 2017). A variety of DNA viruses manipulate cell cycle to achieve a cellular condition which is favorable to viral replication. Parvovirus induces cell cycle arrest which contributes to the virus-induced cytopathic effect (Chen and Qiu, 2010). Papillomaviruses encode proteins that are able to promote cell proliferation through overcoming G1/S restriction point (R point) (Wasson et al., 2017). Herpesviruses arrest cells in the late G1 phase prior to DNA synthesis (Oster et al., 2005; Paladino et al., 2014). RNA viruses also develop strategies to influence cell cycle in order to create an environment which is beneficial to viral replication (Dove et al., 2006; Kim et al., 2015; Gluck et al., 2017; Ning et al., 2017; Wang Z.Y. et al., 2017).

Coxsackievirus B (CVB) are species of non-enveloped, positive single-stranded RNA viruses that belong to the *Enterovirus* genus in the family of *Picornaviridae* (Garmaroudi et al., 2015; Leveque et al., 2017). The six serotypes of CVB can cause a wide range of illnesses from mild gastrointestinal disorder to severe meningitis or myocarditis, which, in some cases, may progress to cardiomyopathy and heart failure (Alidjinou et al., 2014; Garmaroudi et al., 2015). The genome of CVB encodes four structural proteins (VP1, VP2, VP3, and VP4) (Garmaroudi et al., 2015). These structural proteins are configured into the typical icosahedral capsid. Beside the four structural proteins, CVB also encodes non-structural proteins including RNA-dependent RNA polymerase 3D (3D^pol^) and viral proteases 2A (2A^pro^) and 3C^pro^ (Yajima and Knowlton, 2009). To synthesize viral proteins, the genome of CVB is first translated into a single polypeptide that is cleaved by the viral proteases to generate viral structural and non-structural proteins (Garmaroudi et al., 2015). It is believed that the interaction between viral proteins and cellular machinery plays a key role for the pathogenesis of CVB infection. Extensive studies have been focusing on viral non-structural proteins such as 2A^pro^ and 3C^pro^, since these proteases not only cleave viral polyproteins, but also cleave important cellular proteins such as eukaryotic translation initiation factor 4G (eIF4G) and mitochondrial antiviral-signaling protein (MAVS) (Chau et al., 2007; Feng et al., 2014; Hanson et al., 2016). Thus, CVB infection leads to the disturbance of various cellular machineries including the increased assembly of autophagosomes (Wong et al., 2008; Xin et al., 2015; Wu et al., 2016), up-regulated ubiquitin-proteasome system (UPS) (Luo et al., 2003a), and ER stress (Luo et al., 2018). However, it is not clearly defined how CVB infection would modulate the cell cycle.

A previous study has shown that cell cycle arrest at G1/S phase is favorable for CVB3 replication, while quiescent cells hinder the synthesis of viral proteins (Feuer et al., 2002). The mechanism by which CVB3 blocks cell cycle at G1/S is associated with the reduction of G1 cyclins, cyclin D and E, due to the up-regulated ubiquitin-proteasome proteolysis induced by viral infection (Luo et al., 2003b). While a direct implication of CVB3 in the manipulation of cell cycle has already been demonstrated, whether viral capsid protein VP1 exerts impact on the progression of cell cycle is unknown. In our previous study, we identified that the capsid protein VP1 of CVB3 (hereafter VP1) contains nuclear localization signal and is imported into the nucleus (Wang et al., 2012). In this work, the impact of VP1 on cell cycle was analyzed. Our results show that VP1 arrests cell cycle at G1 phase through up-regulating the expression of heat shock protein 70 (Hsp70). This finding reveals a new role of VP1 in facilitating the replication of CVB3.

## Materials and Methods

### Ethics Statement

All animals were housed in biosafety level 2 containment facilities and cared for in compliance with the regulation on animal care and use of the Harbin Medical University. All the experimental procedures applied to animals were approved by the Ethics Committee of the Harbin Medical University.

### Mice

Balb/c mice were provided by the Laboratory Animal Center, Harbin Medical University. Newborn mice at the age of 1–3 day after birth were used in this study. Mice were euthanized and cardiomyocytes were prepared as described previously (Ehler et al., 2013; Sherry, 2015). Cardiomyocytes were cultured overnight in growth medium containing 10% fetal bovine serum (FBS). Cells were then transfected with pEGFP-VP1 or control vector pEGFP-C1 for 24 h. Cell cycle was analyzed by flow cytometry.

### Cell Culture

HeLa cells were cultured in Dulbecco’s modified Eagle medium (DMEM) (Life Technologies, Carlsbad, CA) supplemented with 10% heat-inactivated FBS (Biological Industries, Israel), penicillin (100 U/ml), and streptomycin (0.1 mg/ml). Cells were incubated in 5% CO_2_ at 37°C and passaged every 24–48 h.

### Virus

Coxsackievirus B3 Woodruff strain was recovered from plasmid pMKS1, which contains the full-length genomic cDNA of CVB3 (Tong et al., 2011). Viruses were propagated in HeLa cells. Virus titer was determined by TCID_50_ as described previously (Zhong et al., 2008). To determine the interaction between CVB3 replication and cell cycle, sub-confluent cells were infected with 1 multiplicity of infection (MOI) of CVB3. After 1 h of absorption, the medium was removed and fresh medium containing 10% FBS was added. Cells were collected at various time points of post-infection (p.i.) for cell cycle analysis.

### Transfection

pcDNA3.1-EGFP-VP1 (designated as pEGFP-VP1) was constructed from pcDNA3.1-EGFP (designated as pEGFP-C1) as described previously (Wu et al., 2014). HeLa cells were transfected with pEGFP-VP1 or pEGFP-C1 using Lipofectamine 2000 (Invitrogen, Carlsbad, CA), according to the manufacturer’s instructions. Briefly, cells were cultured to 70% of confluence in 6-well plates and transfected with transfection mix (8 μg of the plasmid mixed with 10 μl of liposome in DMEM). After 4 h of incubation at 37°C, the transfection mix was removed and fresh medium with 10% FBS and antibiotics were added. Cells were harvested 24 h after transfection for further analysis. Each transfection was performed in triplicate.

### Reverse Transcription and Real-Time Quantitative PCR (RT-qPCR)

Total RNA was extracted by TRIzol (Invitrogen) according to the instructions of the manufacturer. RNA was dissolved in nuclease-free water and was quantified by Nanodrop 2000 spectrophotometer (Thermo Fisher, Waltham, MA). About 1 μg of each total RNA preparation and 4 μl of 5× TransScript All-in-One SuperMix (TransGen, Beijing, China) were used in a total of 20 μl reverse transcription system. Quantitative PCR (qPCR) was carried out on a LightCycler 96 (Roche, Basel, Switzerland) using TransStart Top Green qPCR SuperMix (TransGen, Beijing, China). Amplification of the cDNA was performed in triplicate, using 10 μl 2 × TransStrat Top Green qPCR SuperMix, 0.4 μl of each specific primer (10 μM), and 1 μl of cDNA. PCR program consisted of 5 min activation at 94°C, followed by 40 cycles of 94°C for 5 s, 55°C for 10 s, and 72°C for 10 s. Expression of the glyceraldehyde phosphate dehydrogenase (GAPDH) was used for the normalization of all target RNAs. The relative changes of gene expression were determined by the 2^–ΔΔ^*^Ct^* methods (Livak and Schmittgen, 2001). The PCR primers used in this study are listed in Table 1.

**TABLE 1:** Sequence of primers.

**Primer name**	**Sequence 5′→3′**
CVB3 forward	GCACACACCCTCAAACCAGA
CVB3 reverse	ATGAAACACGGACACCCAAAG
VP1 forward	TGGGTAATAACACCACGACAAGC
VP1 reverse	CACTGGGATTCGTAGATGTTTGC
GAPDH forward	TGCACCACCAACTGCTTAGC
GAPDH reverse	GGCATGGACTGTGGTCATGAG

### RNA Sequencing

HeLa cells were transfected with pEGFP-VP1 for 24 h. Control cells were transfected with pEGFP-C1. Total RNA was extracted by RNeasy mini kit (Qiagen, Hilden, Germany). The extracted RNA was qualified and RNA integrity was measured by electrophoresis in 1.4% agarose to ensure that there was no RNA degradation. One microgram of the total RNA from each sample was used to create sequencing libraries using TruSeq RNA Library Prep Kit (Illumina, San Diego, CA). The quality of the cDNA libraries was assessed by Agilent 2100 Bioanalyzer (Agilent, Santa Clara, CA). Finally, the prepared libraries were sequenced by GeneX Health (Beijing, China) using Illumina HiSeq 2500 (Illumina).

The raw data of RNA-sequencing (RNA-seq) were filtered using FastQC to exclude low quality reads and the reads in which unknown bases were more than 10%. Clean reads were aligned and mapped to human reference genome using TopHat (Trapnell et al., 2009) and assembled by Bowtie 1 (Langmead, 2010). Differentially expressed genes between the VP1-transfected and control cells were identified using Cufflink (Trapnell et al., 2013). Genes with a fold change of larger than 1.3 (|log2 (fold change)| > 1.3) and with adjusted *P-*value less than 0.001 were considered as differentially expressed. The primary data were provided in the Supplementary Materials.

### Synchronization of Cells

Cell cycle synchronization was performed by blocking the cell cycle with thymidine (Nghiem et al., 2001; Banfalvi, 2017). HeLa cells were cultured in 6-well plate to 50% confluence. Cells were washed with PBS twice and cultured in the medium containing 2 mM of thymidine for 19 h. Then cells were washed twice with PBS and grown in normal DMEM containing 10% FBS for 9 h to allow cells to re-enter cell cycle. Then another round of cell cycle blocking was carried out by the addition of thymidine for 18 h. Cells were infected with CVB3 1 h before the termination of second round of synchronization. Then cells were cultured in normal medium for another 9 h.

### Flow Cytometry

HeLa cells were seeded in 6-well plate until 1–5 × 10^6^ cells were obtained. Cells were washed with cold PBS twice and fixed with cold 75% ethanol for 1 h or overnight at 4°C. Cells were suspended with 500 μl of cold PBS, followed by the addition of 20 μl RNase A solution (BestBio, Shanghai, China) and incubation at 37°C for 30 min. Cells were stained with 70 μM propidium iodide (BestBio) in the PBS containing 0.01% Nonidet P-40 at dark for 1 h. Cellular DNA content was quantified by a flow cytometer LSR Fortessa (BD Biosciences, San Jose, CA). Data were obtained based on 30,000 events collected by flow cytometry.

### Western Blot

Cells were collected and washed twice with cold PBS. Cells were lysed with lysis buffer [120 mM Tris–HCl (pH 6.8), 5% SDS, 10% 2-mercaptoethanol, 20% glycerol, 0.01% bromophenol blue] and stored at −80°C until analyzed. 10% polyacrylamide gel electrophoresis was performed. Proteins were blotted to PVDF membrane and detected with corresponding primary and HRP-conjugated secondary antibodies. The blots were imaged with FluorChem R system (ProteinSimple, Santa Clara, CA). Primary antibodies against cyclin E, p27, Hsp70, heat shock factor 1 (HSF1), β-actin, GAPDH, and β-tubulin were obtained from Proteintech (Rosemont, IL). A monoclonal mouse anti-enteroviral VP1 antibody (clone 5-D8/1) (Dako, Glostrup, Denmark) was used to detect CVB3 VP1. Polyclonal antibody against 3D^pol^ of CVB3 was prepared in our laboratory. Antibody against pS326-HSF1 was obtained from Abcam (Cambridge, United Kingdom).

### Fluorescence Microscopy

HeLa cells were plated on glass coverslips and transfected with pEGFP-VP1 or pEGFP-C1 for 24 h. Cells were fixed with 4% formaldehyde in dark. Cells were washed twice and stained with 1 μg/ml DAPI in 0.1% PBS-T for 5 min at 37°C in dark. Then, the stained cells were mounted on slides for confocal observation. Fluorescence images were captured by a CV1000 confocal microscope (Yokogawa, Tokyo, Japan).

### Statistical Analysis

All of the experiments are repeated three times. Data were presented as mean ± SD. Statistical analysis was performed by Graphpad prism7.0. Student *t* test was performed. *P* < 0.05 was set as statistical significance.

## Results

### VP1 of CVB3 Arrests Cell Cycle at G1 Phase

**FIGURE 1:**
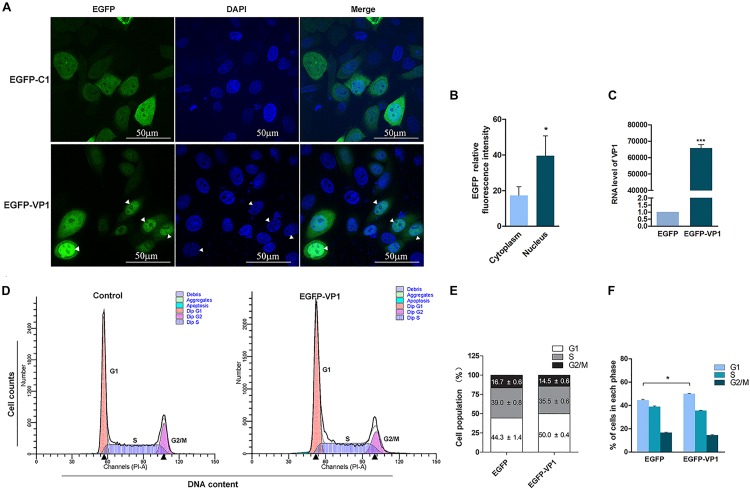
VP1 of CVB3 induces cell cycle arrest at G1 phase. **(A,B)** HeLa cells were transfected with pEGFP-VP1 or pEGFP-C1 for 24 h. The expression of VP1 was determined by fluorescence microscopy (**A**, lower panel; arrow heads indicate the nuclei). The relative fluorescence intensity of EGFP was quantified **(B)**. The RNA level of VP1 was determined by RT-PCR **(C)**. **(D–F)** HeLa cells were transfected with pEGFP-VP1 for 24 h. Control cells were transfected with pEGFP-C1. Cell cycle was analyzed by flow cytometry **(D)**. Cell population distributed in each phase of the cell cycle was compared between the cells expressing VP1 and control cells **(E,F)**. *n* = 3. ^*^P < 0.05, ^∗∗∗^*P* < 0.01.

Our previous study showed that VP1, one of the capsid proteins of CVB3, contains nuclear localization signal, which allows it to be imported into the nucleus (Wang et al., 2012). To further investigate whether VP1 disturbs cell cycle, HeLa cells were transfected with pEGFP-VP1 for 24 h, and cell cycle was measured by flow cytometry. Control cells were transfected with pEGFP-C1. As shown in Figure 1, EGFP was evenly distributed in both cytoplasm and nucleus in the control cells transfected with pEGFP-C1 (Figure 1A, upper panel). VP1 was localized in both cytoplasm and the nucleus, while the accumulation of VP1 in the nucleus was obvious (lower panel of Figures 1A,B). Cell population at G1 phase was significantly increased in the cells expressing VP1 (Figures 1D–F), indicating that VP1, the structural component of CVB3, disturbs the progression of cell cycle.

### VP1 of CVB3 Induces Cell Cycle Arrest in Primary Cardiomyocytes

**FIGURE 2:**
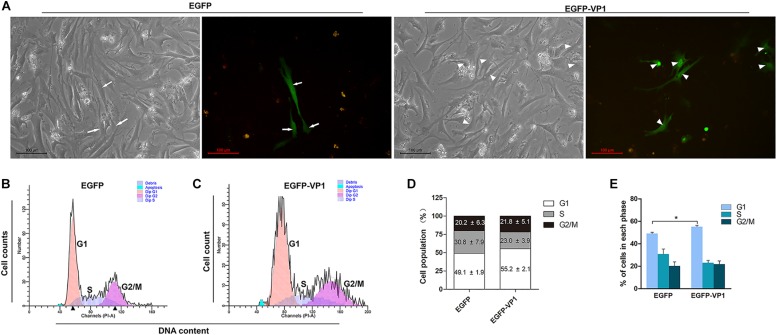
VP1 of CVB3 induces G1 arrest in cadrdiomyocytes. **(A)** Cardiomyocytes were isolated from the hearts of neonatal Balb/c mice. Cells were transfected with pEGFP-C1 or pEGFP-VP1 for 24 h and viewed by fluorescence microscope. **(B,C)** Cell cycle was determined by flow cytometry. **(D,E)** Cell population distributed in each phase of the cell cycle was compared between the cells expressing VP1 and control cells. *n* = 3. ^*^P < 0.05.

Coxsackievirus B3 preferentially infects children and young adults with the consequence of myocarditis and cardiomyopathy (Garmaroudi et al., 2015). Since HeLa cells are highly proliferating cancer cells with features different from the cardiomyocytes, which have limited proliferation ability after birth (Zebrowski et al., 2017). Thus, we asked the question whether VP1 of CVB3 disturb the cell cycle of cardiomyocytes. To this end, primary cardiomyocytes extracted from the hearts of newborn Balb/c mice were transfected with pEGFP-C1 or pEGFP-VP1, and cell cycle was determined. As shown in Figure 2, EGFP was localized evenly in the entire cell, while VP1 was accumulated in the nucleus (Figure 2A). Cell population in G1 phase was significantly increased in the cardiomyocytes expressing EGFP-VP1, compared with that of the cells expressing EGFP (Figures 2B–E). These data show that VP1 indeed hinders the progression of cell cycle of the cardiomyocytes.

### VP1 Dysregulates the Expression of Cyclin E and p27^Kip1^

**FIGURE 3:**
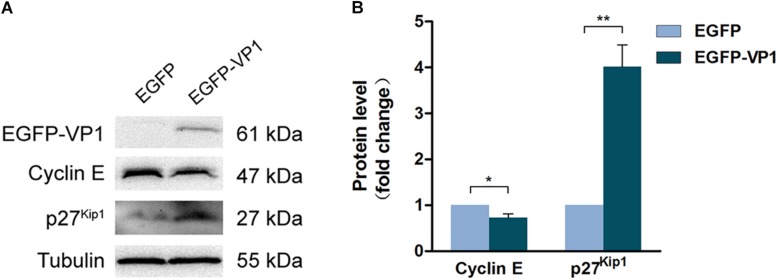
VP1 dysregulates the expression of cyclin E and p27^*kip1*^. **(A,B)** HeLa cells were transfected with pEGFP-C1 or pEGFP-VP1 for 24 h. Cyclin E and p27^*kip1*^ were determined by Western blotting. *n* = 3. ^*^P < 0.05, ^∗∗^*P* < 0.01.

To elucidate the mechanism through which VP1 arrests cell cycle progression, HeLa cells were transfected with the plasmid expressing EGFP-VP1 for 24 h, and the expression of cell cycle regulatory proteins was determined by Western blotting. We found that in the cells expressing VP1 (shown in Supplementary Figure S1), the level of cyclin D1 remained unchanged, while and phosphorylated (at serine 780) retinoblastoma protein (p-Rb), which promotes the progression of G1 (Hassan et al., 2004), was increased (Supplementary Figure S1A). p21 and p53, the Cdk inhibitors (Scully et al., 2018), were down-regulated (Supplementary Figure S1B). Other Cdk inhibitors such as p15, p16, and p57 remained unchanged (Supplementary Figure S1B). However, cyclin E level was decreased, while p27^Kip1^, one of the inhibitors of Cdks, was increased (Figures 3A,B). These results suggest that cyclin E and p27^Kip1^ play a key role for G1 arrest in the cells expressing VP1.

### VP1 Arrests Cell Cycle Through Up-Regulating the Expression of Heat Shock Protein 70

**FIGURE 4:**
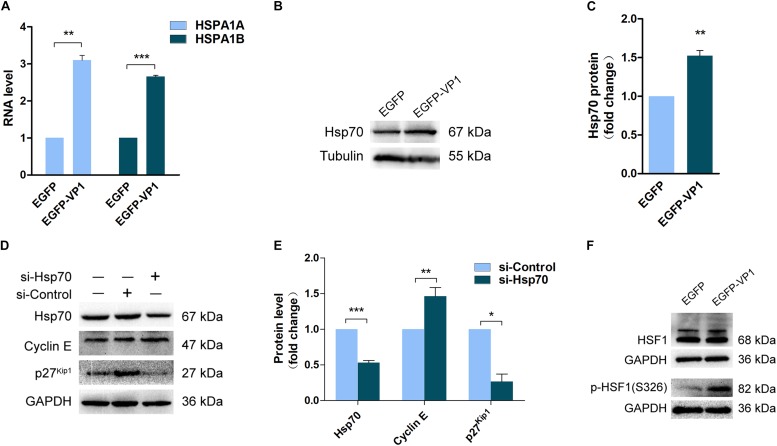
Up-regulated Hsp70 in the cells expressing VP1 results in the dysregulation of cyclin E and p27^*kip1*^. HeLa cells were transfected with pEGFP-C1 or pEGFP-VP1 for 24 h. **(A)** The expression of Hsp70 was determined by RNA-sequencing. **(B,C)** The expression of Hsp70 was verified by Western blotting. **(D,E)** HeLa cells were transfected with si-Hsp70 for 24 h. Cell lysate was prepared and cyclin E, p27^*kip1*^, and Hsp70 were determined by Western blotting. **(F)** HeLa cells were transfected with pEGFP-C1 or pEGFP-VP1 for 24 h, HSF1 and p(S326)-HSF1 were determined by Western blotting. *n* = 3. ^*^P < 0.05, ^∗∗^*P* < 0.01, ^∗∗∗^*P* < 0.001.

To reveal the mechanism by which VP1 manipulates cell cycle, we investigated the gene expression profile of the cells transfected with pEGFP-VP1 by RNA-seq. The results show that there was almost no change in the gene expression profile in the cells expressing VP1, except five genes which were dysregulated. (The results of RNA-seq were provided as Supplementary Material.) Among the genes with altered expression levels are *HSPA1A* (GenBank: KY500386.1) and *HSPA1B* (GenBank: KY500397.1), which encode the chaperone protein heat shock protein 70-1 (Hsp70-1) and Hsp70-2, respectively. The expression of Hsp70 was confirmed by RT-qPCR (Figure 4A) and Western blotting (Figures 4B,C).

The concomitantly increased expression of Hsp70-1 and Hsp70-2 in the cells expressing VP1 suggests that Hsp70 may play a critical role in cell cycle control. To reveal the role of Hsp70 in G1 arrest, Hsp70 was knocked down by siRNA, and the expression of cyclin E and p27^Kip1^ was determined. Knockdown of Hsp70 resulted in the significant accumulation of cyclin E, while p27^Kip1^ was reduced (Figures 4D,E). These data suggest that the increased expression of Hsp70 in the cells expressing VP1 at least contributes to the reduced level of cyclin E and the accumulation of p27^Kip1^, which lead to G1 arrest.

To answer this question how the expression of Hsp70 is regulated, we determined the phosphorylation status of HSF1, the transcription factor that directly controls the transcription of heat shock proteins (Zheng et al., 2016). The activation of HSF1 is controlled by phosphorylation. Studies have shown that CVB3 activates calcium/calmodulin-dependent protein kinase II (CaMKII), which phosphorylates HSF1 at serine 230 (Qiu et al., 2016; Wang F. et al., 2017). In this study, we determined the phosphorylation of HSF1 at serine 326, which also promotes the activation of HSF1 (Chou et al., 2012). As shown in Figure 4F, the phosphorylation level of HSF1 at serine 326 was significantly elevated in the cells overexpressing VP1, indicating that the increased expression of Hsp70 is induced by the activation of HSF1.

### CVB3 Replication Induces Cell Cycle Arrest at G1 Phase

**FIGURE 5:**
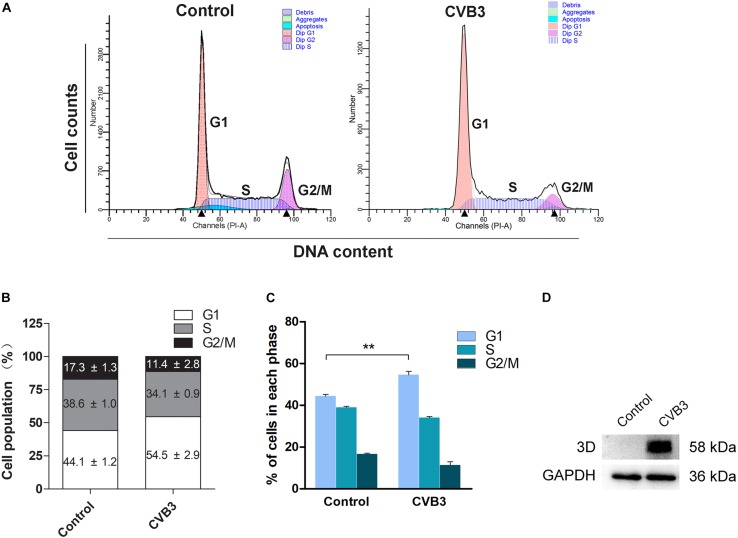
Coxsackievirus B3 infection induces G1 arrest. HeLa cells were cultured in 6-well plate to 60% confluence and infected with CVB3 at 1 MOI for 24 h. Mock-infected cells were set as control. **(A)** Cell cycle was determined by flow cytometry at 24 h of post-infection. **(B)** Cell population distributed in each phase of the cell cycle was calculated. **(C)** Cell population was compared between mock-infected and CVB3-infected cells. **(D)** The 3D^*pol*^ of CVB3 was determined by Western blotting. *n* = 3. ^∗∗^*P* < 0.01.

We further studied if the infection of CVB3 also dysregulates cell cycle progression. HeLa Cells were infected with CVB3 (MOI = 1) for 24 h. Cell cycle was analyzed by flow cytometry. As shown in Figure 5, cells infected with CVB3 also showed significantly increased population of cells at G1 phase.

### CVB3 Infection Dysregulates Cyclin E and p27^Kip1^

**FIGURE 6:**
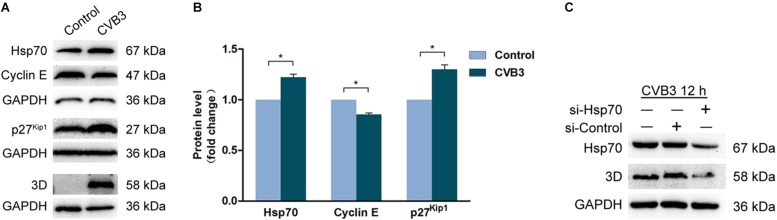
Coxsackievirus B3 infection dysregulates the expression of Hsp70, cyclin E, and p27^kip1^. **(A,B)** HeLa cells were cultured in 6-well plate to 60% confluence and infected or mock-infected with CVB3 at 1 MOI for 24 h. Cell lysates were prepared and subjected to the analysis of Western blotting. **(C)** HeLa cells were transfected with si-Hsp70 for 24 h and infected with CVB3 at MOI of 1 for 12 h. Cell lysates were prepared and subjected to the analysis of Western blotting. *n* = 3. ^*^P < 0.05. si-Control: Control si-RNA.

To investigate the mechanism by which CVB3 induces G1 arrest, cells were infected with CVB3 (MOI = 1) for 24 h, the protein levels of Hsp70, cyclin E, and p27^*kip1*^ were determined by Western blotting. In the cells infected with CVB3, Hsp70, and p27^Kip1^ were markedly increased, while cyclin E was decreased (Figures 6A,B). These data show that the dysregulated Hsp70, cyclin E, and p27^Kip1^ contribute to G1 arrest induced by CVB3 infection. These data also imply that VP1 at least contributes to G1 arrest during CVB3 infection.

It has been demonstrated that Hsp70, which is up-regulated in CVB3-infected cells, shows beneficial impact on the stability of viral genome (Qiu et al., 2016). To confirm the role of Hsp70 in CVB3 infection, viral replication was determined in the cells with the knockdown of Hsp70. As shown in Figure 6C, knockdown of Hsp70 dramatically reduced the level of viral 3D^pol^, indicating that Hsp70, which is up-regulated in CVB3-infected cells, facilitates viral replication.

### Cell Cycle Arrest Promotes CVB3 Replication

**FIGURE 7:**
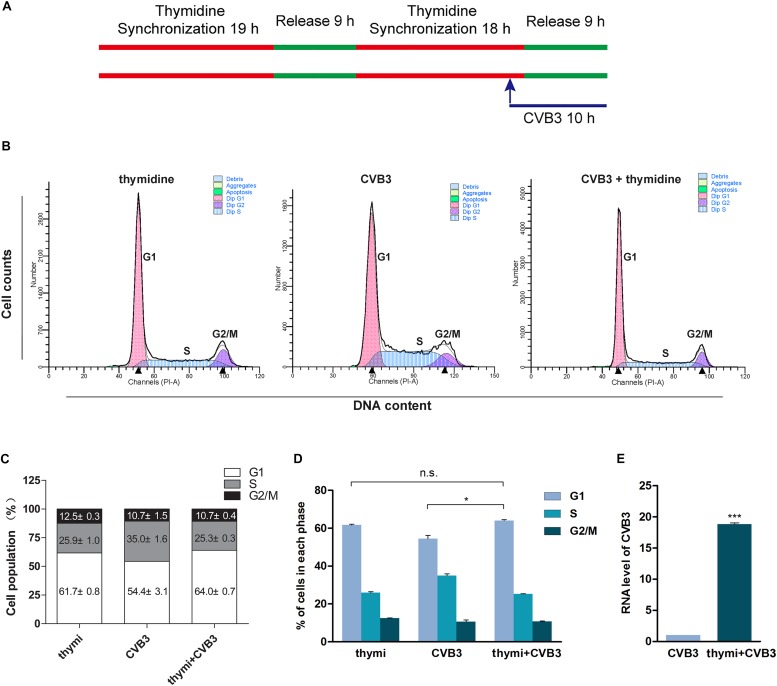
G1 arrest facilitates the replication of CVB3. **(A)** HeLa cells were synchronized at G1 by theaddition of thymidine and cells were infected with CVB3. **(B)** Cell cycle was analyzed by flow cytometry. **(C)** Cell population distributed in the various phases of cell cycle was calculated. **(D)** Cell population in G1 phase was compared between CVB3-infected cells with and without treatment of thymidine. **(E)** Viral RNA of CVB3 was determined by RT-qPCR. *n* = 3. ^*^P < 0.05, ^∗∗∗^*P* < 0.001. n.s.: no significant.

It has been reported that CVB3 replication is promoted by cell cycle inhibitors which induce cell cycle arrest at G1/S phase (Feuer et al., 2002). To confirm that the arrest of G1 phase facilitates CVB3 replication, HeLa cells were synchronized at G1 phase through the addition of thymidine and infected with CVB3 (MOI = 1) (Figure 7A). Compared with non-synchronized cells, cells synchronized at G1 phase (Figures 7B–D) showed significantly increased level of CVB3 genome (Figure 7E). These data indicate that G1 arrest facilitates CVB3 replication.

## Discussion

To create a favorable cellular environment is the common strategy used by viruses. In the present study, we demonstrate that VP1, one of the capsid proteins of CVB3, induces cell cycle arrest at G1 phase through up-regulating Hsp70.

G1/S transition is crucial for cell cycle control (Fisher, 2016). In mammalian cells, the presence of growth factors promotes the expression of cyclin D, which binds and activates Cdk4/6. The activated Cdk4/6 initiates the phosphorylation of Rb, the cell cycle repressor (Moser et al., 2018). The p-Rb releases a fraction of the transcription factor E2F, which promotes the transcription of the genes including cyclin E and A. These cyclins couple with Cdk2 which functions as a positive feedback to trigger the switch from hypo- to hyper-phosphorylation of Rb. At this point, cell commits to complete the current division cycle even without the presence of growth factors (Bracken et al., 2004). Fully released E2F induces the production of proteins required for S-phase entry. In mammalian cells, the critical point between late G1 and the entry of S phase is defined as the R point (Moser et al., 2018).

We previously showed that the VP1 of CVB3 contains nuclear localization signal, and the nuclear translocation of VP1 disturbs cell cycle (Wang et al., 2012). This study further evaluated the influence of VP1 on cell cycle and revealed the underlying mechanism. To study the mechanism of the disturbed cell cycle, we determined the regulatory proteins associated with the control of G1 phase. We found that cells expressing VP1 showed up-regulated p-Rb, decreased levels of p21^Cip^ and p53. These data seem to suggest that the expression of VP1 could promote G1/S transition rather than arrest cell cycle at G1 phase. The hyper-phosphorylation of Rb is critical for G/S transition (Johnson and Skotheim, 2013; Fisher, 2016). However, we have no evidence concerning the phosphorylation status of Rb through determining a single phosphorylation site (serine 780) of this protein. Thus, data from the present study are not sufficient for us to reach the conclusion whether or not R point passage is achieved in the cells expressing VP1. Evidence has shown that at early G1 phase, Rb is mono-phosphorylated by cyclin D-Cdk4/6. At the later stage of G1 phase, cyclin E-Cdk2 complex inactivates Rb by hyper-phosphorylation (Narasimha et al., 2014; Fisher, 2016). These observations suggest that cyclin E, in combination with Cdk2, plays a crucial role for the hyper-phosphorylation of Rb and the entry of S phase. Moreover, studies in recent years support the concept that the mechanism, which controls S-phase entry when cells are under stress, might be very distinct from that of the normal cycling cells (Johnson and Skotheim, 2013).

Our data demonstrate that the level of cyclin E was reduced in the cells expressing VP1, while p27^kip1^ was accumulated. These results suggest that lack of cyclin E and/or the accumulation of p27^kip1^ are sufficient to induce G1 arrest. In consistent with our findings, previous study has shown that the accumulation of cyclin E occurs after the passage of R point and before the entry of S phase (Ekholm et al., 2001), indicating that adequate level of cyclin E is critical for S phase entry. Evidence also shows that E2F is not sufficient to induce G1/S transition without cyclin E (Duronio and O’Farrell, 1995). Therefore, declined cyclin E level in the cells expressing VP1 would at least contribute to G1 arrest.

We further explored the mechanism leading to the dysregulated cyclin E and p27^kip1^ in the cells expressing VP1. The results of RNA-seq demonstrate that the expression of Hsp70 was up-regulated in the cells overexpressing VP1. Since Hsp70, which is translated in 5′-cap independent mechanism, is also up-regulated in the cells infected with CVB3 (Qiu et al., 2016), we presumed that the increased expression of Hsp70 could be responsible for the dysregulated cell cycle control proteins in the cells expressing VP1. And indeed, the dysregulated cyclin E and p27^Kip1^ were related with the up-regulation of Hsp70 in the cells expressing VP1. Knockdown of Hsp70 with siRNA resulted in the accumulation of cyclin E and the reduction of p27^Kip1^. These data indicate that the up-regulated Hsp70 causes the decrease of cyclin E and the accumulation of p27^Kip1^, leading to G1 arrest.

Hsp70-1 and Hsp70-2 belong to the Hsp70 family with only one amino acid difference (Leppa et al., 2001). As important molecular chaperone, Hsp70 plays critical role in protein folding, membrane translocation of proteins, and protein degradation via proteasome pathway (Nitika, and Truman, 2017). The expression of Hsp70 is often induced by cellular stress such as infection, ischemia, inflammation, and exposure to oxidants (Shamovsky and Nudler, 2008; Radons, 2016). Previous studies have demonstrated that up-regulated Hsp70 during CVB3 infection promotes viral replication (Helmbrecht et al., 2000; Qiu et al., 2016; Wang F. et al., 2017). CVB3 hijacks the host cell to produce viral RNA and proteins by shutting down the cap-dependent translation of cellular proteins, while the translation of viral proteins relies on the internal ribosome entry site (IRES) in the 5′ untranslated region (UTR) of viral genome (Bonderoff and Lloyd, 2008; Ullmer and Semler, 2018). However, the translation of Hsp70 depends on IRES rather than cap-dependent (Qiu et al., 2016). This ensures that its synthesis is not interfered by CVB3 infection.

In agreement with the previous report (Qiu et al., 2016), we also show that the phosphorylation of HSF1 is likely responsible for the elevated expression of Hsp70 in the cells expressing VP1. HSF1, which is the key transcription regulator that binds the heat shock response element upstream of the promoter of *HSPA* (the gene coding Hsp70) (Chou et al., 2012). In addition, it is implicated that the activation of HSF1 is also regulated by Hsp70. HSF1 basally interacts with Hsp70, and this interaction was interrupted by stress when misfolded proteins compete with HSF1 for the binding of Hsp70 (Zheng et al., 2016). Phosphorylation, while as a positive factor that facilitates the activation of HSF1, seems not play a critical role (Zheng et al., 2016). It has been shown that Hsp70 interacts with P1, the capsid precursor of poliovirus and CVB1 (Macejak and Sarnow, 1992). Based on the reported studies and our results, we postulate that, in addition to the up-regulated phosphorylation of HSF1, VP1 of CVB3 might interfere with the interaction between HSF1 and Hsp70 through binding to either one of these proteins, leading to the release and activation of HSF1.

Cyclins and Cdk inhibitors are degraded through UPS (Cao et al., 2011; Ning et al., 2017), while Hsp70 plays an important role in UPS-mediated protein degradation (Fernandez-Fernandez et al., 2017). UPS is also promoted and utilized by CVB3 infection (Luo et al., 2003b; Gao et al., 2008). Therefore, similar to the finding that CVB3 promotes the degradation of cyclin D (Luo et al., 2003b), it is possible that the reduced abundance of cyclin E is the result of its up-regulated degradation through UPS in the cells expressing VP1 or infected with CVB3. However, this mechanism cannot explain the accumulation of Cdk inhibitor p27^Kip1^. A recent study demonstrated that the ubiquitination of p27^Kip1^ by the ubiquitin-conjugating enzyme UBCH7/UBE2L3 stabilizes p27^Kip1^ while other Cdk inhibitors are not affected (Whitcomb et al., 2019), suggesting that there is an intricate mechanism which controls p27^Kip1^. In consistent with our finding, the up-regulation of Hsp70 and p27^Kip1^ has been reported in the cells with G1 arrest induced by radiation (Seo et al., 2006).

During the infection of CVB3, viral proteases cleave various cellular proteins that are vital for the normal function of the host cell (Chau et al., 2007; Feng et al., 2014; Hanson et al., 2016). Thus, the increased expression of Hsp70 could be one of the protective responses of the host cells to maintain proteostasis under the stress of viral infection (Xu et al., 2019). Although the gene expression profile remains almost undisturbed in the cells expressing VP1, as a viral component, VP1 is very likely identified as a foreign protein by the host cell, which triggers the stress response including the increased expression of Hsp70. Moreover, the nuclear localization of VP1 might directly or indirectly influence the expression of limited genes including *HSPA1A* and *HSPA1B*. Although we show that the increased level of Hsp70 is likely due to the elevated phosphorylation level of HSF1, which directly promotes the transcription of Hsp70, it remains unknown how VP1 could impact the phosphorylation of HSF1.

Unlike our previous findings (Wang et al., 2012), here, we show that the cells expressing VP1 induced cell cycle arrest at G1 phase rather than S phase. The inconsistence is due to different experimental settings. Our previous study primarily focused on the nuclear localization character of VP1. We emphasized the difference between the outcomes yielded by VP1 and VP1_*H220T*_, a mutated VP1 which loses the nuclear localization capability, in order to confirm that the nuclear translocation of VP1 disturbs cellular functions. Therefore, the cell cycle test was controlled by the cells expressing VP1_*H220T*_ and normal cells, which had not been treated with the control plasmid pEGFP-C1. Later we realized that VP1_*H220T*_ may not be a proper control for the cell cycle evaluation, because it may disturb the nuclear function. Our microscopic observation showed that VP1_*H220T*_ binds to the nuclear membrane and blocks the nuclear pores (Wang et al., 2012). In this study, with an optimized experimental setting, we demonstrate that VP1 of CVB3 actually caused G1 arrest.

## Conclusion

We demonstrate that the capsid protein VP1 of CVB3 induces cell cycle arrests at G1 phase through the increased expression of Hsp70, which is up-regulated by the elevated phosphorylation of HSF1. The increased Hsp70 results in the reduced level of cyclin E and the accumulation of p27^Kip1^. Furthermore, G1 arrest facilitates the replication of CVB3.

## Data Availability

The raw data supporting the conclusions of this manuscript will be made available by the authors, without undue reservation, to any qualified researcher.

## Ethics Statement

All animals were housed in biosafety level 2 containment facilities and cared for in compliance with the regulation on animal care and use of the Harbin Medical University. All the experimental procedures applied to animals were approved by the Ethics Committee of the Harbin Medical University.

## Author Contributions

WZ and ZZ designed the study. YaoW, SZ, and YC performed the experiments. TW constructed the expression plasmids. CD performed flow cytometry. WX generated the antibody against 3D^pol^ of CVB3. XF, CQ, and YanW prepared the primary cells of the mice. WZ, YaoW, and ZZ analyzed the data. WZ and ZZ drafted the manuscript. Remaining authors provided substantial support during the implementation of this study. All authors read and approved the final version of the manuscript.

## Conflict of Interest Statement

The authors declare that the research was conducted in the absence of any commercial or financial relationships that could be construed as a potential conflict of interest.
