# Supplementary material for: The Capsid Protein VP1 of Coxsackievirus B Induces Cell Cycle Arrest by Up-Regulating Heat Shock Protein 70
Source: Front Microbiol. 2019 Jul 17;10:1633. doi: 10.3389/fmicb.2019.01633 (PMC6653663; doi:10.3389/fmicb.2019.01633)
Supplement: Supplementary file 2 [file Data_Sheet_1.docx]

**The capsid protein VP1 of coxsackievirus B induces cell cycle arrest by up-regulating heat shock protein 70**

Yao Wang^1^, Shuoxuan Zhao^1^, Yang Chen^2^, Tianying Wang^2^, Chaorun Dong^3^, Xiaoman Wo^1^, Jian Zhang^1^, Yanyan Dong^1^, Weizhen Xu^2^, Xiaofeng Feng^1^, Cong Qu^1^, Yan Wang^2^, Zhaohua Zhong^2*^, Wenran Zhao^1*^

^1^ Department of Cell Biology, Harbin Medical University, 157 Baojian Road, Harbin 150081, China

^2^ Department of Microbiology, Harbin Medical University, 157 Baojian Road, Harbin 150081, China

^3^ Northern Translational Medicine Research Center, Harbin Medical University, 157 Baojian Road, Harbin 150081, China

**Supplementary Material**

**Materials used for Western blot**

Antibody against phosphorylated retinoblastoma protein was obtained from Abcam (pSer780). Antibodies against p15, p16, p21, p53, p57 were obtained from Proteintech (Wuhan, China).

**Supplementary Figure S1**


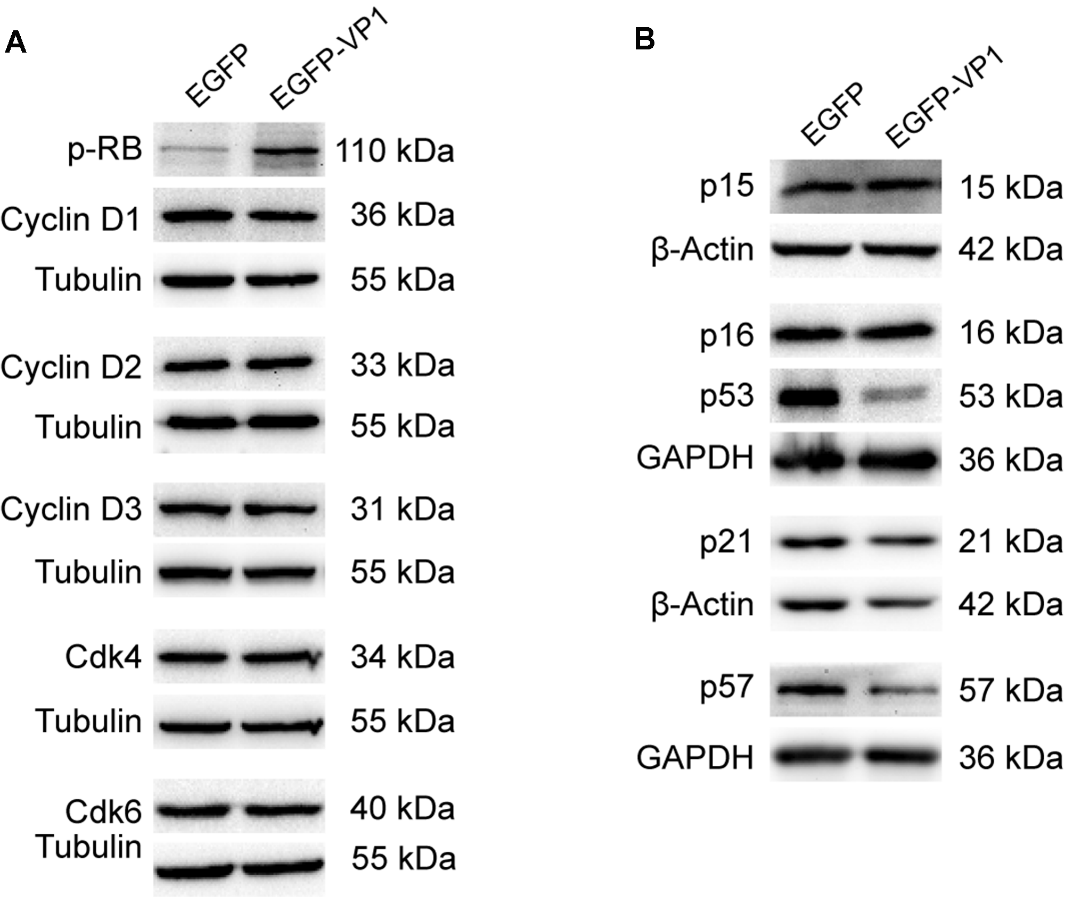


**Supplementary Figure S1. The expression of the cell cycle regulatory proteins in the cells expressing VP1**. HeLa cells were transfected with pEGFP-C1 or pEGFP-VP1 for 24 h. Cell lysate was prepared and subjected to the analysis of Western blotting (A and B).
